# Supplementary material for: Content-rich biological network constructed by mining PubMed abstracts
Source: BMC Bioinformatics. 2004 Oct 8;5:147. doi: 10.1186/1471-2105-5-147 (PMC528731; doi:10.1186/1471-2105-5-147)
Supplement: Additional File 2 — The original results of the above study (non-essential files are deleted to keep the file size under the limit set by BMC bioinformatics). [file 1471-2105-5-147-S2.bz2 › chilibotAdditionalFile2/dip30/608ID1701053E10990/html/CSF3R_CSF3.html]

 


 **CSF3R** and **CSF3** 
  
Found 215 abstracts in PubMed, retrieved 30.  
 

 What does Google say? 
 PDF only 
| .edu only 

---

**Interactive relationship** (e.g. stimulation, inhibition, etc)

**Stimulatory relationship**- However, these mature bloodstream neutrophils express the GCSF  [ **CSF3** ]  receptor GCSFR  [ **CSF3R** ] , presenting a significant and specific clearance mechanism of circulating GCSF  [ **CSF3** ]  that increases with time.  Ref: 12488547 Mol Pharmacol, 2003
- Granulocyte colony stimulating factor  [ **CSF3** ]  regulates neutrophil production by binding to a specific receptor, the granulocyte colony stimulating factor  [ **CSF3** ]  receptor  [ **CSF3R** ] , expressed on cells of the granulocytic lineage.  Ref: 12524421 J Biol Chem, 2003
- Granulocyte colony stimulating factor  [ **CSF3** ]  G CSF  [ **CSF3** ]  induces proliferation, maturation, and functional activities of myeloid progenitors and mature neutrophils through a specific receptor, the G CSF  [ **CSF3** ]  R  [ **CSF3R** ] .  Ref: 12586631 Blood, 2003
**Neutral relationship**- Titration microcalorimetry experiments using a soluble murine granulocyte colony stimulating factor  [ **CSF3** ]  receptor  [ **CSF3R** ]  construct show that SB 247464 binds to the extracellular domain of the receptor in a zinc ion dependent manner.  Ref: 12524421 J Biol Chem, 2003
- Studies in cell lines have established that conserved tyrosines Tyr704, Tyr729, Tyr744, Tyr764 within the cytoplasmic domain of G CSF  [ **CSF3** ]  receptor G CSF  [ **CSF3** ]  R  [ **CSF3R** ]  contribute significantly to G CSF  [ **CSF3** ]  induced proliferation, differentiation, and cell survival.  Ref: 12468431 Blood, 2003
- Replacement of the gp130 domains by the corresponding domains of the related GCSF  [ **CSF3** ]  receptor either restores weak LIF responsiveness D4 GCSFR  [ **CSF3R** ] , leads to constitutive activation of gp130 D5 GCSFR  [ **CSF3R** ]  or results in an inactive receptor D6 GCSFR  [ **CSF3R** ] .  Ref: 12047380 Eur J Biochem, 2002
- G CSF  [ **CSF3** ]  activates a receptor of the hematopoietin receptor superfamily, the G CSF  [ **CSF3** ]  receptor G CSF  [ **CSF3** ]  R  [ **CSF3R** ] , which subsequently triggers multiple signaling mechanisms.  Ref: 12687405 Rev Physiol Biochem Pharmacol< MedlineTA>Rev Physiol Biochem Pharmacol, 2002
- G CSF  [ **CSF3** ]  affects these cells after binding to its specific receptor, G CSF  [ **CSF3** ]  R  [ **CSF3R** ] , which exists in seven isoforms.  Ref: 12660437 Biol Neonate, 2003

**Non-interactive relationship** (e.g. studied together, co-existance, homology, etc.)

- Two models, the human growth hormone human growth hormone receptor hGH hGHR 1 2 and the granulocyte colony stimulating factor  [ **CSF3** ]  granulocyte colony stimulating factor  [ **CSF3** ]  receptor  [ **CSF3R** ]  GCSF  [ **CSF3** ]  GCSFR  [ **CSF3R** ]  2 2 complexes, were used for modeling.  Ref: 11944966 Gen Comp Endocrinol, 2002
- The progenipoietins ProGPs are a family of genetically engineered chimeric proteins that contain receptor agonist activity for both fetal liver tyrosine kinase 3 and the granulocyte colony stimulating factor  [ **CSF3** ]  receptor  [ **CSF3R** ] .  Ref: 12406682 Protein Expr Purif, 2002
- These findings suggest that tyrosines in the cytoplasmic domain of G CSF  [ **CSF3** ]  R  [ **CSF3R** ] , although dispensable for G CSF  [ **CSF3** ]  induced colony growth, recruit signaling mechanisms that regulate the maintenance and outgrowth of myeloid progenitor cells.  Ref: 12468431 Blood, 2003
- Potential redundancy among members of the CCAAT enhancer binding protein C EBP family in myeloid cells is indicated by the ability of C EBPbeta to replace C EBPalpha in vivo, by the expression of granulocyte colony stimulating factor  [ **CSF3** ]  receptor  [ **CSF3R** ]  G CSFR on C EBPalpha cell lines, and by our finding that as with C EBPalpha estrogen receptor C EBPalpha ER, either C EBPbeta ER or C EBPdelta ER can induce terminal granulopoiesis in 32D cl3 cells.  Ref: 11929766 Blood, 2002
- An activating mutation in the transmembrane domain of the granulocyte colony stimulating factor  [ **CSF3** ]  receptor  [ **CSF3R** ]  in patients with acute myeloid leukemia.  Ref: 12203110 Oncogene, 2002
- On the other hand, Ras activation was necessary for all receptor mediated proliferation with the exception of G CSF  [ **CSF3** ]  R  [ **CSF3R** ] .  Ref: 12032860 Oncogene, 2002
- Long term follow up of granulocyte colony stimulating factor  [ **CSF3** ]  receptor  [ **CSF3R** ]  mutations in patients with severe congenital neutropenia implications for leukaemogenesis and therapy.  Ref: 12588357 Br J Haematol, 2003
- Our cell level model suggests that ligand depletion may be reduced in vitro by decreasing the endosomal affinity of endocytosed GCSF  [ **CSF3** ]  GCSFR  [ **CSF3R** ]  complexes, matching experimental findings.  Ref: 12488547 Mol Pharmacol, 2003
- CCAAT enhancer binding proteins are required for granulopoiesis independent of their induction of the granulocyte colony stimulating factor  [ **CSF3** ]  receptor  [ **CSF3R** ] .  Ref: 11929766 Blood, 2002
- Suppressor of cytokine signaling 3 is recruited to the activated granulocyte colony stimulating factor  [ **CSF3** ]  receptor  [ **CSF3R** ]  and modulates its signal transduction.  Ref: 12133942 J Immunol, 2002
- Here, we investigated how individual G CSF  [ **CSF3** ]  R  [ **CSF3R** ]  tyrosines affect G CSF  [ **CSF3** ]  responses of primary myeloid progenitors.  Ref: 12468431 Blood, 2003
- SB 247464 induces internalization of granulocyte colony stimulating factor  [ **CSF3** ]  receptor  [ **CSF3R** ]  on intact cells, consistent with a mechanism involving receptor oligomerization.  Ref: 12524421 J Biol Chem, 2003
- Signaling mechanisms coupled to tyrosines in the granulocyte colony stimulating factor  [ **CSF3** ]  receptor  [ **CSF3R** ]  orchestrate G CSF  [ **CSF3** ]  induced expansion of myeloid progenitor cells.  Ref: 12468431 Blood, 2003
- 0.01 and G CSF  [ **CSF3** ]  r  [ **CSF3R** ]  = 0.875, P <.  Ref: 12569227 Med Sci Sports Exerc, 2003
- We also demonstrated that tyrosine phosphorylation of the Src kinases Hck and Lyn occurs following G CSF  [ **CSF3** ]  stimulation of cells expressing WT G CSF  [ **CSF3** ]  R  [ **CSF3R** ] , but that Hck is not phosphorylated in mutant G CSF  [ **CSF3** ]  R  [ **CSF3R** ]  transfectants.  Ref: 12586631 Blood, 2003
- G CSF  [ **CSF3** ]  R  [ **CSF3R** ]  mRNA was only detected in two myeloid cell lines.  Ref: 12088257 Tumori< MedlineTA>Tumori, 2003
- The transcription factor C EBP alpha regulates early steps of normal granulocyte differentiation since mice with a disruption of the C EBP alpha gene do not express detectable levels of the granulocyte colony stimulating factor  [ **CSF3** ]  receptor  [ **CSF3R** ]  and produce no neutrophils.  Ref: 11978795 J Biol Chem, 2002
- The current study demonstrates that activated Ras enhances the ability of C EBP alpha to transactivate the granulocyte colony stimulating factor  [ **CSF3** ]  receptor  [ **CSF3R** ]  promoter and a minimal promoter containing only C EBP DNA binding sites.  Ref: 11978795 J Biol Chem, 2002
- These cytokines transduce their biological signal via the Epo receptor EpoR and G CSF  [ **CSF3** ]  receptor G CSF  [ **CSF3** ]  R  [ **CSF3R** ]  into the cell.  Ref: 12088257 Tumori< MedlineTA>Tumori, 2002
- Our data provide evidence that the carboxy terminal region of G CSF  [ **CSF3** ]  R  [ **CSF3R** ]  plays a role in the phagocytosis of IgG coated particles.  Ref: 12586631 Blood, 2003
- Here, we formulate a mathematical model that describes these cell level GCSF  [ **CSF3** ]  GCSFR  [ **CSF3R** ]  dynamics and correlate the effect of these endocytic trafficking processes to ligand depletion in an in vitro culture.  Ref: 12488547 Mol Pharmacol, 2003
- In approximately 20% of SCN cases, a truncation mutation is found in the cytoplasmic region of the granulocyte colony stimulating factor  [ **CSF3** ]  receptor  [ **CSF3R** ]  G CSFR.  Ref: 12672695 Blood, 2003
- CD34 cells from 45 patients with myelodysplastic syndrome MDS and MDS acute myeloid leukaemia MDS AML were observed by flow cytometry for the expression of granulocyte colony stimulating factor  [ **CSF3** ]  receptor  [ **CSF3R** ]  G CSFR.  Ref: 12670333 Br J Haematol, 2003
- IL 10 Milenia Biotec, Bad Nauheim, Germany, gammaINF Diaclone, Besancon, France and G CSF  [ **CSF3** ]  R  [ **CSF3R** ]  &.  Ref: 12788309 Cytokine, 2003
- Here we review how these mechanisms contribute to the specific responses of hematopoietic cells to G CSF  [ **CSF3** ]  and how perturbations in the function of the G CSF  [ **CSF3** ]  R  [ **CSF3R** ]  are implicated in various types of myeloid disease.  Ref: 12687405 Rev Physiol Biochem Pharmacol< MedlineTA>Rev Physiol Biochem Pharmacol, 2003
- Real time PCR analysis revealed that TPT augmented the expression not only of CD18 but also of components of superoxide generating NADPH oxidase, p47phox, 2.7 fold, and p67phox, 2.0 fold, and of granulocyte colony stimulating factor  [ **CSF3** ]  receptor  [ **CSF3R** ]  G CSFR, 3.0 fold.  Ref: 12788061 Biochem Biophys Res Commun, 2003
- Messenger RNA expression of granulocyte colony stimulating factor  [ **CSF3** ]  receptor  [ **CSF3R** ]  isoforms in the fetus.  Ref: 12660437 Biol Neonate, 2003
- The following tests did not distinguish patients with CMCA from other SDS patients severity of peripheral cytopenia, fetal hemoglobin levels, percentage of marrow CD34 cells, colony growth from marrow CD34 cells, cluster to colony ratio, marrow stromal function, percentage of marrow apoptosis cells, and granulocyte colony stimulating factor  [ **CSF3** ]  receptor  [ **CSF3R** ]  expression.  Ref: 12135662 Exp Hematol, 2002
- We generated G CSF  [ **CSF3** ]  R  [ **CSF3R** ]  deficient mice and transduced their bone marrow cells with tyrosine null mutant m0, single tyrosine add back mutants, or wild type WT receptors.  Ref: 12468431 Blood, 2003
- Characterization of granulocyte colony stimulating factor  [ **CSF3** ]  receptor  [ **CSF3R** ]  expressed on human lymphocytes.  Ref: 12100165 Br J Haematol, 2002
- In all SCN patients studied no abnormalities of granulocyte colony stimulating factor  [ **CSF3** ]  receptor  [ **CSF3R** ]  G CSFR gene were detected by polymerase chain reaction single strand conformation polymorphism analysis and sequence analysis.  Ref: 12422946 Hiroshima J Med Sci, 2002
- METHODS AND STUDY DESIGN The expression of EpoR and G CSF  [ **CSF3** ]  R  [ **CSF3R** ]  mRNA was analyzed with reverse transcription polymerase chain reaction RT PCR.  Ref: 12088257 Tumori< MedlineTA>Tumori, 2002
- We evaluated the involvement of Syk kinase, essential in mediating phagocytic signals by Fcgamma receptors, in G CSF  [ **CSF3** ]  induced phagocytosis, using murine myeloid 32D cells transfected with wild type WT human G CSF  [ **CSF3** ]  R  [ **CSF3R** ]  hG CSF R or with a G CSF  [ **CSF3** ]  R  [ **CSF3R** ]  mutant truncated at cytoplasmic amino acid 715.  Ref: 12586631 Blood, 2003
- To elucidate the function of Cb2 in myeloid leukemia cells, a set of in vitro experiments was carried out using 32D G CSF  [ **CSF3** ]  R  [ **CSF3R** ]  granulocyte colony stimulating factor  [ **CSF3** ]  receptor  [ **CSF3R** ]  cells transfected with exon 1B exon 2 Cb2 complementary DNA and a myeloid cell line carrying a virus insertion in Cb2 ie, NFS 78.  Ref: 11929767 Blood, 2002
- To assess the biologic significance of these findings, we introduced PML and PML RARalpha into interleukin 3 IL 3 dependent Ba F3 cells expressing the chimeric receptor composed of extracellular domain of G CSF  [ **CSF3** ]  R  [ **CSF3R** ]  and cytoplasmic domain of gp130, in which gp130 mediated growth is essentially dependent on STAT3 activity.  Ref: 12506013 Blood, 2003
- While information exists about the distribution of these isoforms in hematopoietic cells and placenta, G CSF  [ **CSF3** ]  R  [ **CSF3R** ]  isoforms on non hematopoietic fetal tissues have not been described.  Ref: 12660437 Biol Neonate, 2003
- Seven genes, including interleukin 1 receptor antagonist, IL 1receptor type II, insulin like growth factor binding protein 4, transforming growth factor beta, granulocyte colony stimulating factor  [ **CSF3** ]  receptor  [ **CSF3R** ] , leukemia inhibitory factor receptor, and soluble IL 4 receptor were selected as probable candidate genes for the trabecular bone sparing effect of estrogen, as the mRNA levels of these genes were highly correlated to the trabecular BMD.  Ref: 12469912 J Bone Miner Res, 2002
- Bone marrow derived primitive CD34 and CD33 CD34 progenitor cells from SCN patients evolving to AML, all with mutations in the granulocyte colony stimulating factor  [ **CSF3** ]  receptor  [ **CSF3R** ]  G CSFR gene, demonstrated normal cell survival.  Ref: 12763135 Exp Hematol, 2003
- Recent studies have shown that point mutations in granulocyte colony stimulating factor  [ **CSF3** ]  receptor  [ **CSF3R** ]  G CSFR are involved in the pathogenesis of severe congenital neutropenia SCN and in the transformation of SCN to acute myelogenous leukemia AML.  Ref: 12012328 J Cell Physiol, 2002
- The G CSF  [ **CSF3** ]  R  [ **CSF3R** ]  mutant lacks the immunoreceptor tyrosine based activation motif ITAM, putative binding site for Syk.  Ref: 12586631 Blood, 2003
- Thus in the human fetus, G CSF  [ **CSF3** ]  R  [ **CSF3R** ]  isoform I is the predominant form expressed on non hematopoietic and hematopoietic tissues.  Ref: 12660437 Biol Neonate, 2003
- Selective binding and oligomerization of the murine granulocyte colony stimulating factor  [ **CSF3** ]  receptor  [ **CSF3R** ]  by a low molecular weight, nonpeptidyl ligand.  Ref: 12524421 J Biol Chem, 2003
- The carboxy terminal region of the granulocyte colony stimulating factor  [ **CSF3** ]  receptor  [ **CSF3R** ]  transduces a phagocytic signal.  Ref: 12586631 Blood, 2003
- To this end, we have developed selective amplifier genes SAGs that encode chimeric proteins that are a fusion of granulocyte colony stimulating factor  [ **CSF3** ]  receptor  [ **CSF3R** ]  and the steroid binding domain.  Ref: 12666183 J Gene Med, 2003
- Different signals are mediated by distinct regions of the cytoplasmic domain of G CSF  [ **CSF3** ]  R  [ **CSF3R** ] , but the precise role of each region has not yet been fully clarified.  Ref: 12586631 Blood, 2003
- We have studied the characterization of granulocyte colony stimulating factor  [ **CSF3** ]  receptor  [ **CSF3R** ]  G CSFR in human lymphocytes.  Ref: 12100165 Br J Haematol, 2002
